# Adaptive strategies in architecture and allocation for the asymmetric growth of camphor tree (*Cinnamomum camphora* L.)

**DOI:** 10.1038/s41598-024-72732-1

**Published:** 2024-09-30

**Authors:** Chenbing Guo, Yonghong Hu, Jun Qin, Duorun Wu, Hanbing Leng, Hongbing Wang

**Affiliations:** 1 Shanghai Collaborative Innovation Center of Plant Germplasm Resources Development, College of Life Sciences, Shanghai Normal University, No. 100 Guilin Road, Xuhui District, Shanghai, 200234, China; 2 Shanghai Chenshan Botanical Garden, No. 3888 Chenhua Road, Songjiang District, Shanghai, 201602, China; 3 Shanghai Chenshan Plant Science Research Center, Chinese Academy of Sciences, No. 3888 Chenhua Road, Songjiang District, Shanghai, 201602, China; 4 Shanghai Botanical Garden, No. 1111 Longwu Road, Xuhui District, Shanghai, 200231 China

**Keywords:** Asymmetric architecture, Camphor tree (*Cinnamomum camphora*), Biomass distribution, Resource allocation, Root-shoot balance, Urban ecology, Plant stress responses

## Abstract

The stability-related asymmetry in roots, trunk, and crown is always found as a typical effect of biomechanical design under heterogeneous stimulus environment. However, it appears to be a conflict between the biomechanical principle and the source-sink distance of nutrient allocation strategies when the orientational asymmetry occurs. Adaptive growth strategies associated with biomass and nutrient allocation remain to be explored. This study used both the minirhizotron and harvest methods to test the effect of trunk inclination of camphor trees (*Cinnamomum camphora*) and found that the asymmetry coefficient of root biomass was − 0.29, showing more root biomass distributed on the other side of trunk inclination. This side had larger surface area and volume of fine roots, the smaller in diameter and the larger in length of the first level roots, higher leaf total nitrogen (TN) and slightly higher root TN content, higher activities of antioxidant enzymes SOD, POD, and CAT in leaves, and lower soluble sugar and protein. The biomass, morphological and physiological characteristics suggest that trees may follow both the biomechanical design and source-sink distance of nutrient allocation strategies. The research results expand the connotation of root-shoot balance in the orientational allocation of biomass and physiological responses.

## Introduction

For more than 200 years, many forest ecologists have been interested in the stability-related asymmetries of tree organs. Wind force, one typical dynamic load on the trees, are always found to cause asymmetric architectures in tree crown, trunk, and root system^1,2^. The adaptive growth has been found in multiple tree species worldwide^3^. The asymmetric traits can also be formed because of the directional heterogeneity in sunlight intensity, soil moisture, nutrient contents, sloping terrain, and neighbors^3^. The asymmetrical morphological traits exist not only in natural forests, but also in urban trees. For instance, road foundations severely limit the growth space of tree roots, resulting in asymmetric distribution of root system; and the tree growing close to buildings can be constrained, causing asymmetric crown^4^. The anchorage and stability of trees in urban environments are of particular concern for safety reasons^5–7^. The asymmetrical growth of trees happens in the asymmetric response to the asymmetric environmental stimuli to form the asymmetric architecture of crown, trunk and root system. The adaptive growth patterns have widely been surveyed, analyzed and evaluated to understand the stability of trees.

The asymmetric crown may reduce tree stability^5,6^, but the plant can still maintain equilibrium based on the biomechanical designs, such as well-anchored root system^8–10^, asymmetry of trunk wood structure^11^, and shorter tree in height^12^. It is a biomechanical balance as a result of biologically adaptive growth to realize the root-shoot mechanical balance by modifying internal structures and morphological responses^13^. Under asymmetric stresses, the orientational distribution of below- and above-ground biomass shows an optimal structure morphology^8–10,14,15^, thus improving tree stability^3,16^. Plants can optimize root architecture to resist external stress through the adaptive growth and development of taproots and lateral roots^17^, although root morphology may vary among tree species. Even a single tree species will alter its morphogenesis due to a heterogeneous environment^18,19^. Wind-induced crown often grows towards the leeward side, and the gravity center of crown deviates from the trunk base^20,21^, while the root system transfers more biomass to the opposite side of the asymmetry of crown^22,23^. Unilateral wind loads can significantly influence morphological plasticity, including increased stem section eccentricity, decreased root volume, and reinforcement of the zone of rapid taper in the leeward side^24,25^; and larger diameter of the structural roots of *Larix decidua* and *Picea sitchensis* on the windward side^22,26^. From a biomechanical point of view, trees maintain functional balance depending on the biomechanical design of below- and above-ground biomass allocation. However, conflicting views of biomechanical balance remain. For example, in the stability evaluation of *Picea sitchensis*, some studies have found that the windward side contributes more, while others hold the opposite view^4,21,27,28^, showing biomechanical complexity. It is necessary to explore the causes of physiological responses.

As an adaptive growth pattern, trees can regulate their resource allocation for biomechanical balance following the adaptive mechanism termed thigmomorphogenesis in response to external mechanical stimuli^29,30^. Plant’s asymmetric growth is also reflected in the physiological process at the organ and whole-plant levels, which is related to physiological equilibrium^31,32^. Resource allocation reflects the trade-off in partitioning between below- and above-ground organs^33–35^, which follows the ‘nutrient allocation’ and ‘functional equilibrium’ strategies^13^. More nutrients will be transported to roots at a low level of below-ground resources (e.g. nutrients and water), and conversely, more nutrients are allocated towards crown at a low level of above-ground resources (e.g. light and CO_2_)^36,37^. For instance, plants allocate more nitrogen to leaves to compensate for the reduced photosynthetic rate induced by decreasing stomatal conductance under arid conditions^38^. Due to the heterogeneous soil moisture availability, plants may exhibit asymmetric formation by the different resource allocations^39,40^. As a complex integrated system, plant organs interact via effective transport pathways for carbon, water, nutrients, and signaling molecules^41^. The whole long-distance transport system may influence on functional balance of nutrients (e.g. carbon) by transporting more from lateral branches to the roots on the same side than that on the opposite side based on the source-sink distance of nutrient allocation, i.e. a sink being supplied by the nearest available source^42–44^. For example, more carbon was distributed to roots from the leaves on the same side relative with that on the opposite side under the asymmetric pruning treatment of *Cunninghamia lanceolata*^13^. The architectural connectivity of tree organs depends on their relative orientation, which is critical for the functional balance of nutrients between leaves and roots. However, studies have found that the below- and above-ground structural interactions follow the biomechanical principle, showing an opposite asymmetry pattern, while more nutrients were distributed to the same side of roots-leaves to follow the source-sink distance of nutrient allocation strategy. There seems to be a conflict between the two strategies. Further studies should focus on the analysis of the adaptive strategies in architecture and allocation for asymmetric growth.

In this study, we used the camphor tree (*Cinnamomum camphora* L.) as a test species to identify the adaptive strategies of asymmetric growth in terms of morphological traits, biomass distribution, and nutrient allocation. The camphor tree is an important ornamental species in subtropical evergreen broad-leaved forests and is widely cultivated in East and South Asia^45^. It has the advantages of vigorous vitality, evergreen, and well-developed root systems, which is considered to be an excellent experimental material. Previous studies have found the opposite asymmetric correlation between below- and above-ground biomass distributions of camphor tree^46^. Therefore, this paper aimed to answer the question: How about the adaptive strategies of resource allocation and biomechanical balance when trees are under asymmetric stress? The results will enrich the connotation of root-shoot balance in the orientational allocation of biomass and physiology responses, which will be the basis for future potential root design in silviculture and management of camphor forest.

## Materials and methods

### Study site

**Fig. 1:**
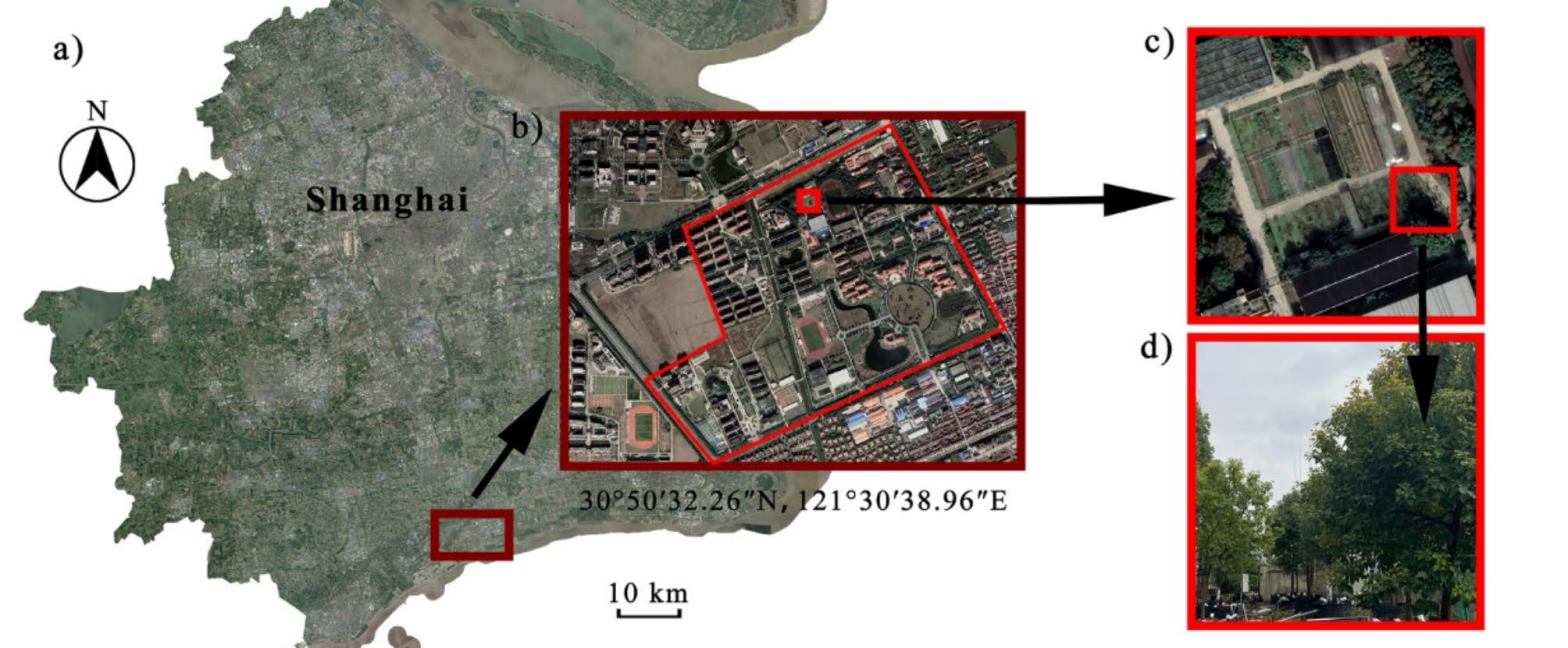
Schematic diagram of planting site. (**a**) The map of Shanghai (downloaded from Google Earth Pro (Google Inc., California, USA)); (**b**) The area where the planting site is located—Fengxian Campus, Shanghai Normal University; (**c**) Top view of the planting site; (**d**) Side view of sample trees.

The study was conducted at the Fengxian Campus, Shanghai Normal University (N 30° 50′ 32.26″, E 121° 30′ 38.96″; Fig. 1), which is a part of the Yangtze River Delta Plain. The average altitude ranges from 3 to 5 m above the sea level in Shanghai. It belongs to a subtropical marine monsoon climate, with an average temperature of 18.1 °C and an average annual rainfall of 1388.2 mm^47^.

### Experimental design

**Fig. 2:**
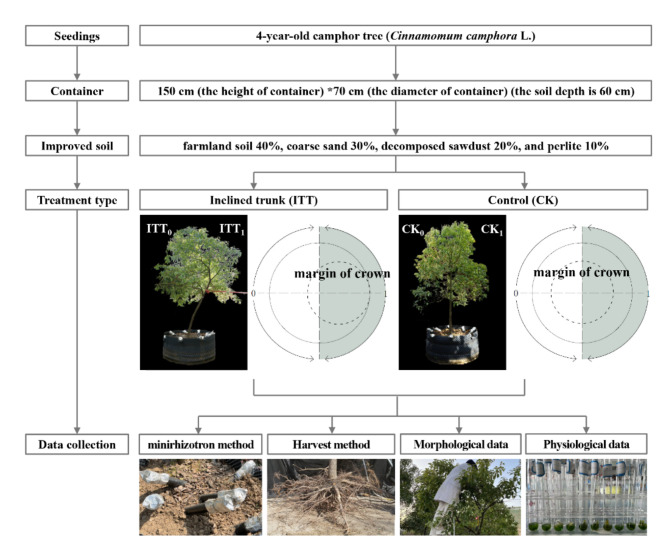
Schematic diagram of experimental design.

The experiment was implemented in April 2019. Three container saplings were planted for treatment and three for control (CK), and the detailed experimental design is outlined in Fig. 2. The experiment was designed as inclined-trunk treatment (ITT), simulating the asymmetric growth of the trees aboveground by artificially bending the trunk to one side with inclination to the ground of approximately 65°. When the sapling was planted, the tree trunk was fixed to a nearby object to make it grow tilted. The control group was designed with no bending trunk. The details were referenced from Wang et al.^45^. To counteract the effects of environmental factors such as solar radiation, wind loading, and soil heterogeneity, we employed a controlled cultivation experimental design considering the following 5 measures: (1) all the 4-year-old saplings were healthy with full crowns, straight stems, and intact soil balls; (2) the containers for planting trees, made of reeled porous PVC and shaped into cylinders, had a diameter of 150 cm and height of 70 cm and were filled with soil up to about 60 cm in depth; (3) the ground is impervious concrete, which can effectively prevent root penetration; (4) all saplings are guaranteed to have uniform initial characteristics, growing space, management, and a similar distance from surrounding buildings and other obstacles; (5) the saplings showed an alternate layout between the treatment and CK in the experimental field.

### Data collection

#### Rhizotron image data on fine roots

Fine roots were measured using the CI-600 In-Situ Root Imager (CID Inc., Camas, WA, USA), from March 2022 to December 2022. Eight minirhizotron tubes with a length of 1 m were buried in the container at a dip angle of 40° to the ground and uniformly distributed. The root system data were collected using CI-600 in March, May, July, September, and December. A reference point was drawn on the upper middle part of each tube end to mark a permanent start-scanning position. The instrument was calibrated before scanning. The Minirhizotron ~ 360° rotating scanner was placed at a vertical depth of approximately 50 cm, and two high-resolution digital images (19.56 cm × 21.59 cm, 100 dpi) were captured at the upper tube depth of 28.59–50.18 cm and lower tube depth of 7–28.59 cm. A total of 96 images were collected each time, and 480 images were taken altogether during the observation period. In the laboratory, the length and diameter of every visible root segment were manually traced and analyzed using the WinRHIZOTron MF 2018a software (Regent Instrument Inc., Québec, Canada). The specific practice of image acquisition and data analysis will be referred to Wang et al. (2022).

#### Harvest method

The data collection commenced from November 15 to December 15, 2022. Root system harvest was performed in two stages: Firstly, superficial roots were first free and bared, followed by progressively deeper roots; Secondly, after the root system was completely dug out, it was cleaned with a high-pressure air gun and hand tools, and then transported to the laboratory^48^. Some broken roots were collected after being uprooted and returned to the original location for measurement and photography. Then, the root system was cut and classified according to the different treatment sides. The leaves and branches were collected in the same way. Then, biomass was determined by drying to constant weight at 80 °C^49^. Meanwhile, 30 leaves on both sides were taken to measure the leaf area and physiological indicators. After removing branches, leaves, and roots, the trunk was divided into three equal parts, and three 5 cm thick wood disks were cut off from each section for scanning and analysis.

#### Morphological data of the aboveground

The morphological indexes of the treatment and CK were measured before harvest. When measuring the branches, it is necessary to grade them. The branches that grow out of the stem were first-level branches; the branches that grow out of the first-level branches were second-level branches, and so on. In this study, the branches were divided into three levels. All the first-level branches were measured, and the second- and third-level branches were sampled in two directions, with ≥ 3 repetitions. Measured indicators include tree height, crown diameter, DBH, base diameter, angle, and length of the sampled branches. The height of saplings in ITT was measured as a vertical line from the highest point of crown to the surface parallel to the trunk base. The angle of sampling branches of each level is the angle between the branch and the upper-level branch, and the angle is less than 180°. After the leaves were scanned, the obtained image was used to calculate the leaf area using ImageJ (National Institutes of Health, Bethesda, Maryland, USA).

#### Physiological data

Before harvest, thirty leaves were randomly sampled from each of the biased and opposite sides of sapling trees and prepared by homogenizing 0.5 g of frozen leaves in 10 ml of extraction buffer (phosphate buffer saline, PBS). Homogenate was centrifuged in the centrifuge at 8,000 rpm and 4 °C for 20 min, and the supernatant was collected as the enzyme extract. Three ml enzyme reaction mixture was reacted to 0.04 ml enzyme extract in the illuminating incubator for 20 min, and then Superoxide dismutase (SOD) activity was measured at 560 nm absorbance as ability to inhibit photochemical reduction of nitro-blue tetrazolium (NBT)^50^. Peroxidase (POD) activity was monitored at 470 nm using guaiacol as the substrate, and catalase (CAT) activity was measured by recording the decrease in absorbance of H_2_O_2_ at 240 nm^51^. A mixture of 2 ml enzyme extract and 2 ml 0.67% thiobarbituric acid solution was heated in 100 °C water bath for 30 min. Sample absorbance was evaluated at 450, 532, and 600 nm using a blank to measure the content of the malondialdehyde (MDA)^52^. Leaf soluble sugar (SS) was determined by standard anthrone colorimetric method^53^. Coomassie brilliant blue method was applied to measure the reference soluble protein (SP) content of leaves^54^. Chlorophyll contents were determined by alcohol extraction method. The measurement of relative conductivity of leaves (LRC) required shaking 0.5 g leaves and 20 ml pure water for 1 h. Following the shock, the conductivity of the solution was measured, and then was boiled at 100 °C for 30 min. When the solution was cooled, the conductivity was measured again and the results were expressed as relative conductivity^55,56^.

Leaves and fine roots were randomly sampled from the biased and opposite sides of each sapling which were used to determine the nitrogen concentration. Sample extraction was performed using sulfuric acid, and followed a similar protocol to that used for soil samples^39^. The K9840 Kjeldahl Nitrogen Analyzer (Hanon Instrument Co. LTD, Jinan, China) was used to measure the total N concentration using the Kjeldahl method.

#### Analysis of morphological data

To analyze the asymmetries of roots and crowns, the directions of asymmetric treatment were defined: *1* represents the biased side of the treated organ with larger mass, that is, the trunk leaning side of ITT; *0* represents the side opposite of *1*. CK_1_ and ITT_1_ were in the same direction. The asymmetry can be explained by crown traits and root traits. The coefficient of asymmetry (CoA) was defined as the ratio of the difference between the two-sided variables to their mean which was used to quantify the bidirectional asymmetry of trees. According to Wang et al. (2022), CoA is calculated as follows:

$$\:CoA=({S}_{1}-{S}_{0})/\stackrel{-}{S}$$

where $\:{S}_{1}$ and $\:{S}_{0}$ represent the data of the *1* and *0* side, respectively. $\:\stackrel{-}{S}$ is the average of $\:{S}_{1}$ and $\:{S}_{0}$. The organs are symmetric when CoA = 0, or asymmetric if CoA ≠ 0. The asymmetric direction is toward the *1* side when CoA > 0, and opposite to the *1* side when CoA < 0.

**Fig. 3:**
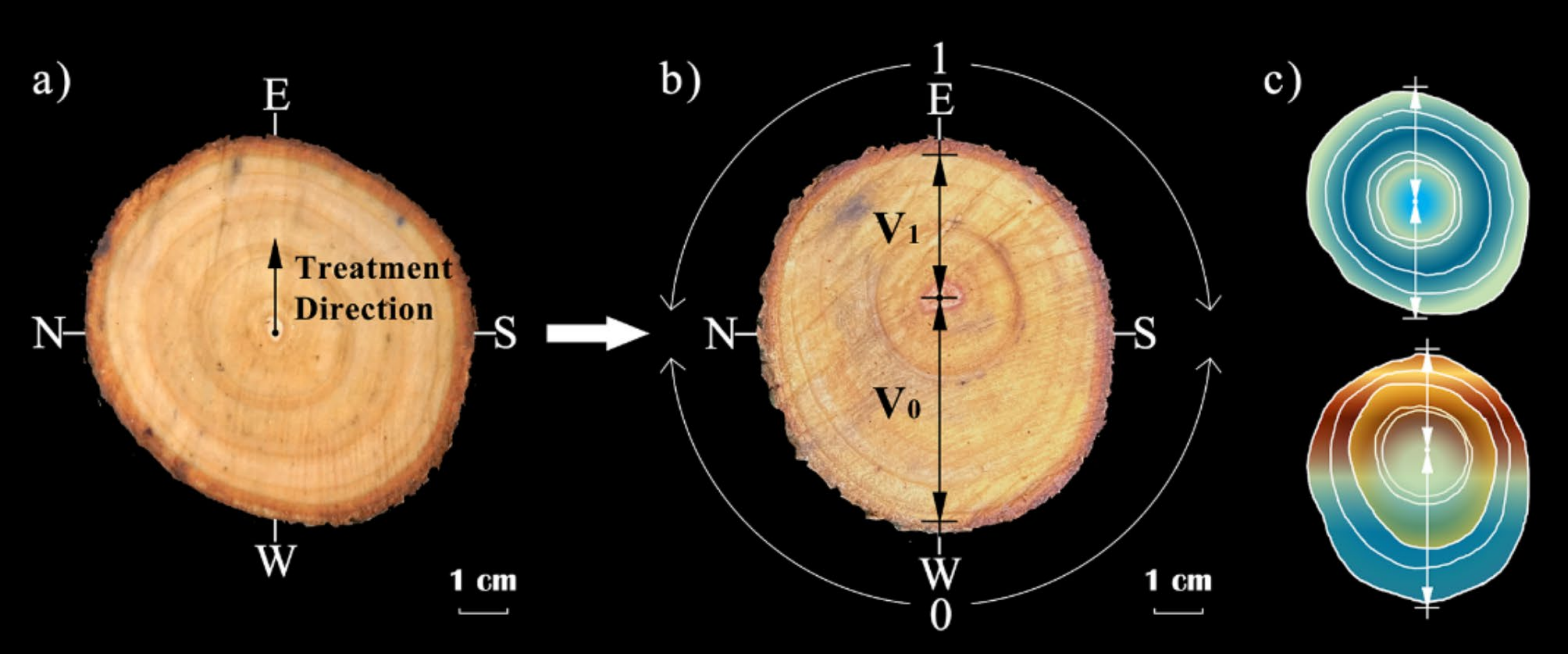
Cross-section schematic diagram of a tree trunk. (**a**) Schematic diagram of treatment direction; (**b**) measurement indicators; (**c**) cross-section of the treatment and control, the upper is CK, the lower is ITT, in which the narrower the red arc spacing, the greater the wood density, and on the opposite the wider the blue arc spacing, the lower the wood density.

There exist different traits and functions from fine root to taproot in root system, so it is necessary to identify and classify roots. This study adopted the method according to the developmental approach^57^. The most proximal roots (taproot) arising from the trunk are typically considered as zero-level roots, while the most distal roots in the system would be the highest order roots (fourth-level roots)^58^. Measurement indicators included root biomass and water content at all levels, and the diameter, length, and angle with the taproot of the five first-level root (roots directly growing on the taproot) were randomly measured on both sides of treatment and control respectively. Specific root length (SRL, i.e., absorptive capability relative to carbon investment) can be used to describe the root traits. It is defined as root length per unit of dry mass. The wood density analysis of the trunk used the method according to Nicoll and Ray, (1996), which was made relative to the annual ring center of the trunk. Dimensions were recorded as follows (Fig. 3): V_1_ = distance above the annual ring center to *1* side, V_0_ = distance above the annual ring center to *0* side. The V_1_/V_0_ ratio compares the thickening in the cross-section. A ratio of 1.0 indicates equal thickening on both sides of the annual ring center, and the higher the ratio, the greater the thickening toward the *1* side relative to the *0* side.

#### Data analysis

All statistical analyses were performed in the SPSS 26.0 statistical software (IBM Corp., Armonk, NY, USA). Before choosing statistical criteria, all data were checked for the normality distribution and Homogeneity of Variances. For the normal distributed and homoscedastic data, one-way ANOVA test was used, and the least significant difference (LSD) method was used for post hoc multiple comparison. For the non-normal data, nonparametric statistical methods, including Kruskal–Wallis test, were applied. Statistical significance was defined as *p* < 0.05 or 0.01.

### The collection and use of any plant materials

Plant samples in the study were purchased. The plant collection and use was in accordance with all the relevant guidelines.

## Results

### Effects of asymmetric growth on the aboveground morphology and biomass

**Fig. 4:**
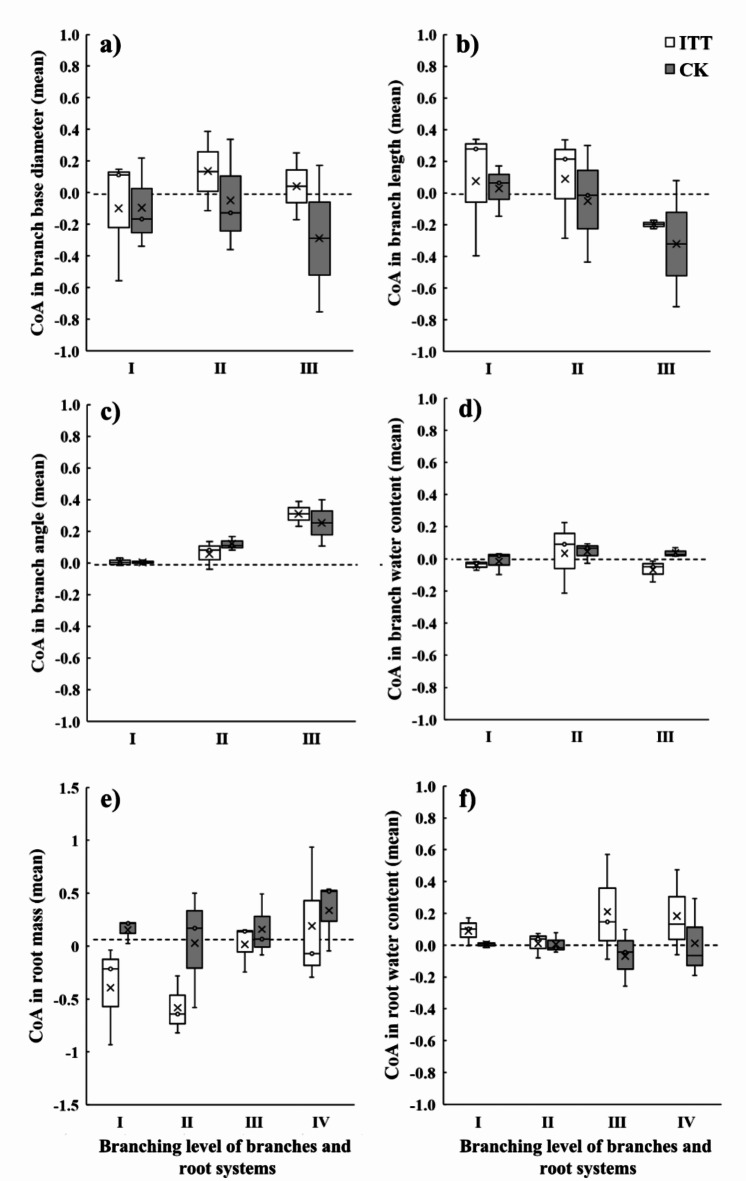
Comparison of CoA of branches and roots at different branching levels in ITT and CK. (**a**–**d**) CoA of branches; (**e**) and (**f**) CoA of roots; I ~ the first-level branch/root; II ~ the second-level branch/root; III ~ the third-level branch/root; IV ~ the fourth-level root.

There were different morphological characteristics of the aboveground organs in response to the asymmetric treatment of ITT (Table 1). Firstly, asymmetric treatment had a significant effect on tree height. The average tree height of ITT was 420.00 cm, which was lower than CK of 497.33 cm (F = 12.047, *p* < 0.05), while ITT had larger DBH than CK. Secondly, in terms of trunk wood density, ITT had the V_1_/V_0_ ratio of 0.76, indicating the significant thickening on the *0* side, while CK had the average V_1_/V_0_ ratio of 1.00, indicating equal thickening on both sides of the annual ring center (F = 22.141, *p* < 0.01). Thirdly, the branch diameter and length in ITT were slightly smaller than that in CK without statistical significance. The CoA of branch base diameter was larger than 0, i.e., the base diameter on the *1* side is greater than that on the *0* side (Fig. 4a). There was a distinction between branches at different levels in ITT and CK. The length of the first- and second-level branches on the *1* side was larger than on the *0* side, and the third-level branch showed an opposite result (Fig. 4b). The CoAs of the angle in ITT and CK showed an increasing trend with the increase of branch level, in which the greater CoA was on the *1* side than on the *0* side in ITT (Fig. 4c). Besides, there was no difference in water content of branches between ITT and CK (Fig. 4d).

**Table 1:** Comparison of physiological characteristics and aboveground morphological indicators of two treatments.

Treatment type	H (cm)	D (cm)	DBH (mm)	CB (g)	LTN (g kg^− 1^)	RTN (g kg^− 1^)	LA (cm^2^)	LRC	LRWC	SOD (U g^− 1^)	POD (U g^− 1^)	CAT (U g^− 1^)	MDA (nmol g^− 1^)	SP (µg g^− 1^)	SS (µg g^− 1^)	Chl (mg g^− 1^)
F/χ^*2*^*-value*	12.047	0.220	2.886	18.446	4.660	1.912	3.988	13.590	4.436	5.516	30.636	15.220	1.684	3.623	15.101	6.289
*p-value*	0.026	0.880	0.165	0.001	0.036	0.174	0.013	0.000	0.218	0.024	0.000	0.000	0.223	0.045	0.003	0.007
CK_0_	497.33 ± 28.37^a^	147.33 ± 17.78^a^	62.83 ± 3.83^a^	2877.52 ± 772.51^b^	8.86 ± 0.46^a^	5.71 ± 1.03^a^	143.99 ± 23.34^b^	0.27 ± 0.01^a^	0.50 ± 0.00^a^	821.05 ± 72.16^a^	812.00 ± 5.65^a^	12.50 ± 0.63^a^	2.77 ± 0.36^a^	81.89 ± 11.25^a^	61.84 ± 0.99^c^	0.23 ± 0.03^b^
CK_1_		142.66 ± 12.05^a^		1894.55 ± 310.85^b^	8.71 ± 0.27^ab^	6.76 ± 1.23^a^	140.50 ± 25.09^b^	0.25 ± 0.03^a^	0.52 ± 0.01^a^	785.96 ± 49.74^a^	807.00 ± 32.52^a^	11.55 ± 1.92^a^	3.06 ± 0.48^a^	77.85 ± 20.61^a^	70.15 ± 1.80^b^	0.21 ± 0.01^b^
ITT_0_	420.00 ± 26.15^b^	145.33 ± 7.57^a^	74.52 ± 11.28^a^	4241.4 ± 852.01^a^	9.02 ± 0.26^a^	5.88 ± 0.62^a^	168.69 ± 23.54^a^	0.15 ± 0.06^b^	0.55 ± 0.05^a^	784.21 ± 22.33^a^	864.22 ± 32.15^a^	12.00 ± 1.76^a^	2.47 ± 0.17^a^	45.15 ± 18.58^b^	67.38 ± 3.75^bc^	0.34 ± 0.10^a^
ITT_1_		139.33 ± 11.59^a^		660.85 ± 276.41^c^	8.08 ± 0.27^b^	5.32 ± 0.77^a^	148.17 ± 16.48^b^	0.29 ± 0.02^a^	0.50 ± 0.01^a^	621.05 ± 44.66^b^	542.88 ± 66.00^b^	7.00 ± 2.69^b^	2.71 ± 0.22^a^	69.01 ± 5.54^ab^	81.74 ± 4.52^a^	0.17 ± 0.03^b^

Notes: H ~ tree height; D ~ crown diameter; DBH ~ diameter at breast height; CB ~ crown (leaves and branches) biomass (dividing the treatment sides with the trunk as a reference line); LTN ~ total nitrogen in leaves; RTN ~ total nitrogen in roots; LA ~ leaf area; LRC ~ leaf relative conductivity; LRWC ~ leaf relative water conten; SOD ~ leaf superoxide dismutase activity; POD ~ leaf peroxidase activity; CAT ~ catalases activity; MDA ~ malondialdehyde content; SP ~ soluble protein; SS ~ soluble sugar; Chl ~ chlorophyll. The lowercase letters (^a^, ^b^ and ^c^) indicate the significant differences among the treatments or directions, *p *＜0.05.

The biomass of aboveground organs, including leaves and branches, was also affected by asymmetric growth. When the trunk was assumed to be approximately straight and used as the reference line, the aboveground biomass on the *0* side was much larger than that on the *1* side in ITT, while there was no difference in CK (Table 1). In contrast, when the reference line was a vertical line passing through the trunk base center, most of the crown body was distributed in the *1* side.

### Effects of asymmetric growth on root morphology, biomass, and water content

**Fig. 5:**
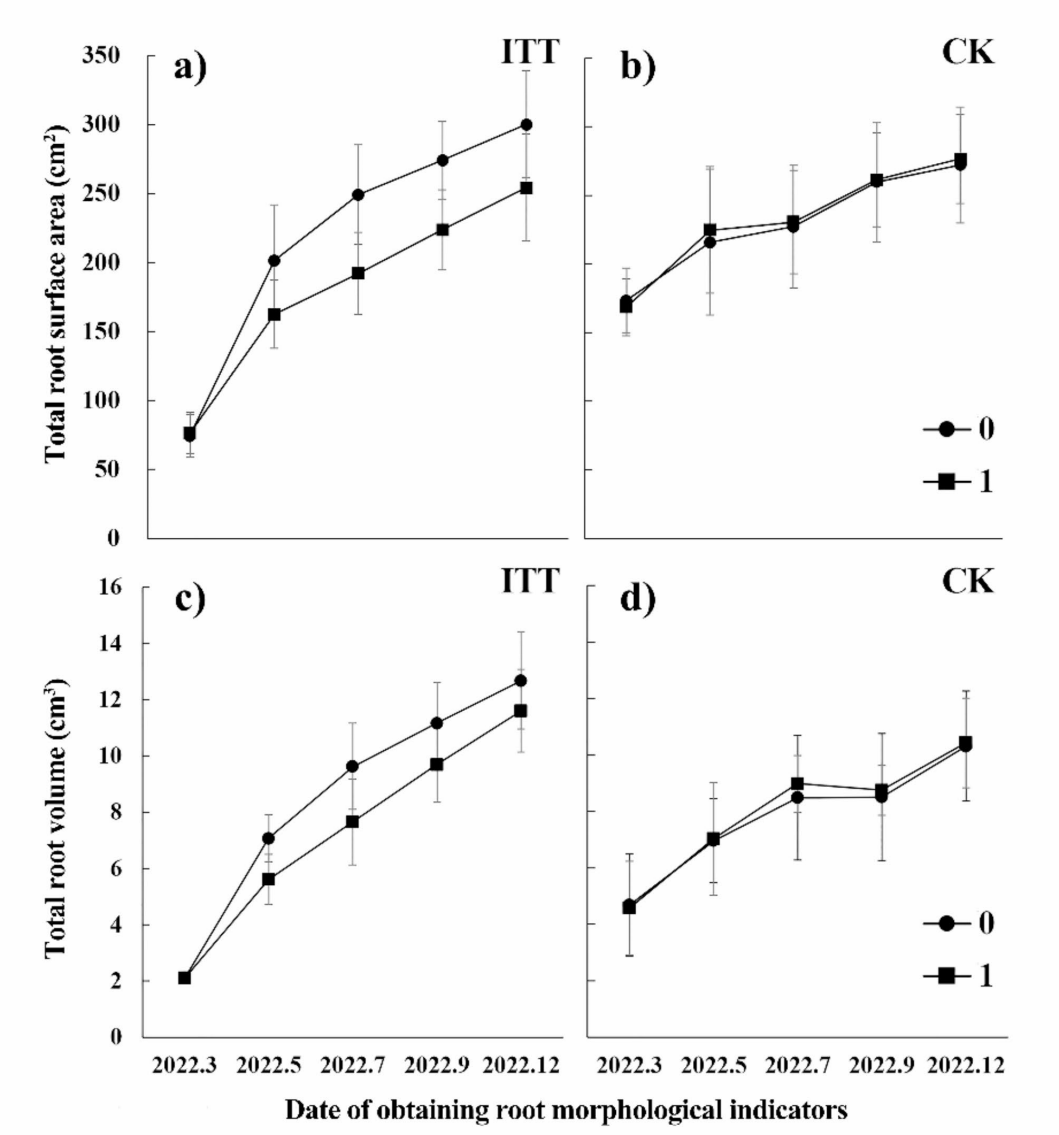
Temporal dynamics of two-sided root morphology in ITT and CK. **a**) and **b**) Total root surface area in ITT and CK; **c**) and **d**) Total root volume in ITT and CK.

**Fig. 6:**
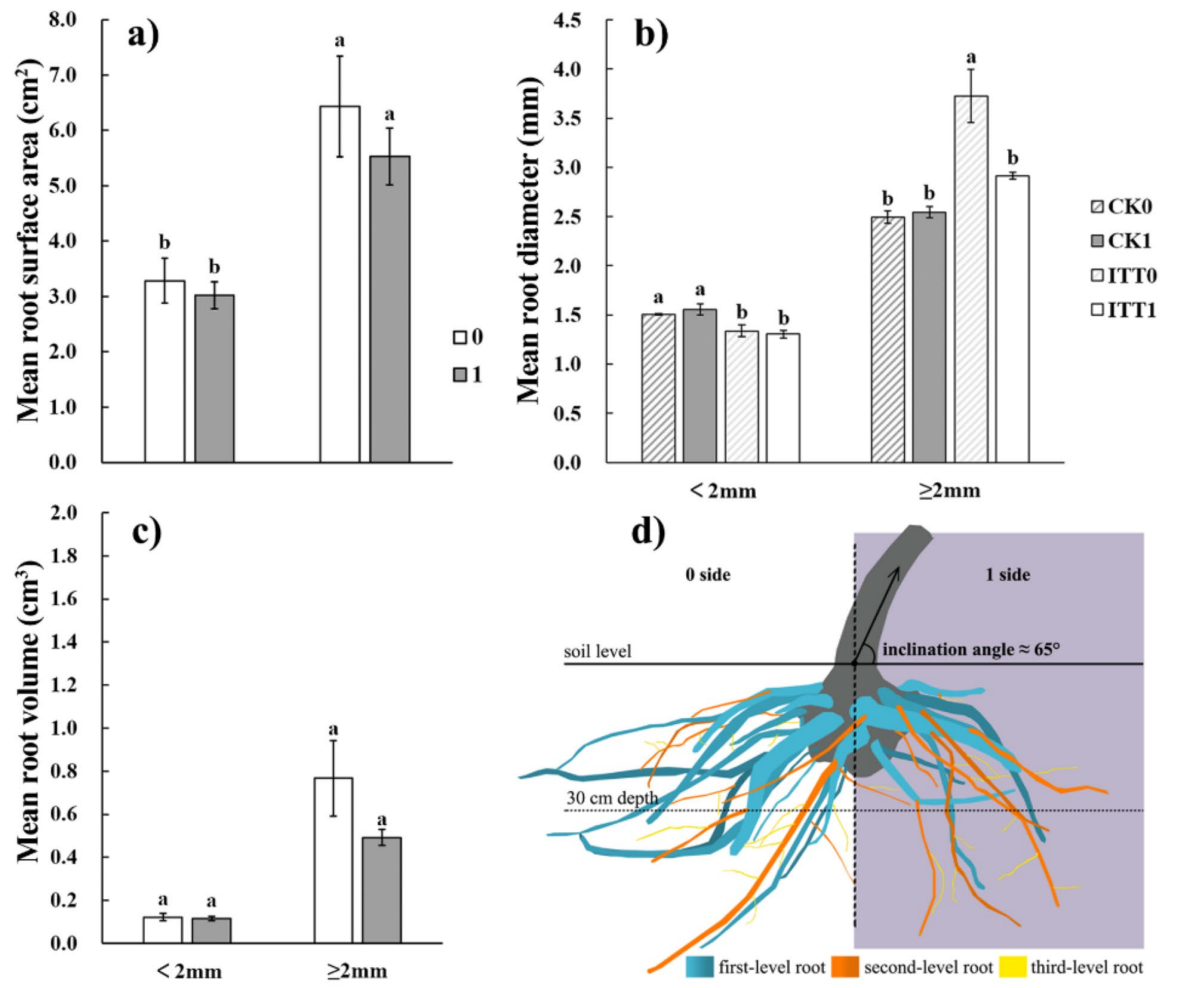
Two-sided comparison of root morphological indicators between two types of roots in ITT and CK. The two types of roots were defined as < 2 mm and ≥ 2 mm in diameter. (**a**)–(**c**) Mean root surface area, diameter and volume in ITT and CK; (**d**) side view of the root system in ITT.

Asymmetric growth resulted in the difference of root biomass distribution on both sides of the sample tree in ITT. The root biomass was 1144.88 g on the *1* side, and 1554.83 g on the *0* side. More root biomass was distributed on the *0* side than that on the *1* side. The CoA of root biomass was − 0.29 in ITT, while that for CK was 0.01. Furthermore, compared with CK, ITT had differences in root biomass at all levels, mainly at the first- and second-levels (Fig. 4e). With the increase of root level, the water content of root system on the *1* side was higher than that on the *0* side (Fig. 4f). From March 2022 to December 2022, the surface area and volume of fine roots maintained an upward trend in ITT and CK. Two-sided comparison analysis found that the root surface area and root volume were larger on the *0* side than that on the *1* side during the test period except March 2022 in ITT (Fig. 5a,c), while no differences were found in CK (Fig. 5b,d). If the roots were divided into two types according to whether the diameter was greater than 2 mm, further analysis of the December 2022 data revealed that the distinctions in surface area and volume of roots between the two sides in ITT presented a trend that the data on the *0* side was larger than that on the *1* side (Fig. 6a,c). The roots of $\:\ge\:$2 mm in diameter showed significantly larger diameters on the *0* side than that on the *1* side in ITT (F = 15.613, *p* < 0.01) (Fig. 6b).

Two-sided first-level roots were measured in ITT and CK, showing some differences in the morphological indicators of the roots randomly sampled (Table 2). The index values of CK were similar on both sides. In ITT, the average base diameter of the first-level roots was 28.25 mm on the *1* side, which was larger than that of 21.46 mm on the *0* side; the first-level root length was 68.74 cm on the *1* side, which was smaller than the *0* side; the angle between first-level root and taproot was 81.26° on the *1* side, which was larger than that on the *0* side; and the SRL of first-level roots was 1.01 on the *1* side, which was smaller compared with that of the *0* side. In general, more first-level roots were found on the *0* side, but in ITT, this type of roots was morphologically larger in diameter and shorter in length on the *1* side. Furthermore, the angle between these roots and taproot was larger, showing a trend of horizontal growth on the *1* side. The side view of root system in ITT can intuitively illustrate these traits (Fig. 6d).

**Table 2:** Comparison of first-level root morphological indicators and SRL. The lowercase letters (^a^ and ^b^) indicate the significant differences among the treatments or directions, *p *＜0.05.

Treatment type	Diameter (mm)	Length (cm)	Angle (°)	SRL (cm/g)
F/χ^*2*^*-value*	9.873	2.314	2.361	0.601
*p-value*	0.020	0.510	0.501	0.632
CK_0_	22.39 ± 8.14^ab^	78.69 ± 13.45^a^	76.65 ± 23.25^a^	1.07 ± 0.46^a^
CK_1_	24.07 ± 6.90^ab^	75.87 ± 10.98^a^	80.64 ± 18.92^a^	1.05 ± 0.32^a^
AT_0_	21.46 ± 7.22^b^	74.48 ± 16.02^a^	76.51 ± 16.99^a^	1.40 ± 0.40^a^
AT_1_	28.25 ± 9.95^a^	68.74 ± 19.22^a^	81.26 ± 12.80^a^	1.01 ± 0.39^a^

### Physiological responses to the asymmetric growth

The asymmetric growth of leaves is mainly reflected in leaf quality, which can be explained by the two-sided comparison of leaf area, relative conductivity, water content, and antioxidant enzyme activity (Table 1). In ITT, the leaf area of the *1* side was significantly smaller than that of the *0* side; leaf water content was higher on the *0* side than that on the *1* side, while there was no difference in CK; leaf relative conductivity was 0.15 on the *0* side, which was lower than that of 0.29 on the *1* side, showing a significant difference (F = 13.590, *p* < 0.01); total nitrogen (TN) content in leaves was significantly greater on the *0* side than on the *1* side, while TN in roots was higher on the *0* side than the other side with non-significant difference. The activities of SOD, POD and CAT on the *0* side were significantly higher than those on the *1* side by 26.25%, 59.19%, and 101.94% (F = 5.516, *p* < 0.05; F = 30.636, *p* < 0.01; F = 15.220, *p* < 0.01, respectively), respectively, while no difference was found in CK; The MDA content was higher on the *1* side than that on the *0* side. The content of SS and SP was opposite to the trend of antioxidant enzyme activities, showing that the SS and SP on the *0* side were lower than that on the *1* side by 34.57% and 17.57% (F = 15.101, *p* < 0.01; F = 3.623, *p* < 0.05, respectively), respectively. Finally, chlorophyll content was significantly higher on the *0* side than that on the *1* side (F = 6.289, *p* < 0.01).

## Discussion

### Asymmetric architecture of trees based on biomechanical balance

As one typical effect of adaptive growth, tree crown deformation and trunk inclination can commonly be found in the windy district, on the sloping terrain, and adjacent to neighbor obstacles, presenting asymmetrical architecture. The aboveground gravity center biases away from the trunk base center, which poses a negative impact on tree stability. The architectural response of root system seems a key in tree anchorage. The root architecture responds to stability-related stresses by increasing the number and length of lateral roots, or producing more sinker roots and tap roots^59–63^. Some studies have found the roots on the windward side contributed more to tree anchorage relative with the roots on the leeward side, which follows the principle of biomechanical design. However, the architectural responses of root system may be species-specific^64,65^. Our study designed the asymmetric scenario of inclined trunk where most of the crown and trunk mass was distributed on the *1* side if the reference line was a vertical line passing through the trunk base center, while more biomass of root system was distributed on the *0* side. There were larger root surface area and root volume on the *0* side than that on the *1* side. The root CoA was − 0.29, showing inverse architectural traits of root system in response to the above-ground asymmetry, and proving that camphor tree follows the principle of biomechanical design to maintain mechanical balance. Furthermore, the roots ≥ 2 mm in diameter showed significantly larger diameters on the *0* side, as well as more first-level roots were found on the side, which also support the biomechanical design in detailed analysis. A similar report has found that European larch and Sitka spruce both had more roots larger than 2 mm in diameter on the windward than that on the leeward^22^. However, a novel finding in this study was that the first-level roots were morphologically larger in diameter, shorter in length, and horizontally oriented growth on the *1* side, which may be related to functional balance between tension component on the one side and compression component on the other side. For example, the windward roots provide tension strength, while the leeward roots provide the compression strength to counteract wind stress for tree anchorage^10,22^. The shallow horizontal roots are believed to improve tree stability^24,66^. There are more and longer roots on the windward side when the thinner roots have higher resistance in tension than the thicker roots^24^. The tensile strength increases with the decrease of root diameter^67^. Better growth of fine roots may represent a feasible strategy to enhance soil resource uptake and plant performance^68^, which supports the adaptive growth traits of root architecture in this study from the perspective of biomechanical design. The orientational relations of the below- and above-ground organs reveal the interdependence between morphology and function, and the spatial relations of root-shoot in biomass distribution.

### Resource allocation in orientational relations of below- and above-ground organs

Optimal partitioning theory is the basis for predicting plant adaptive growth in response to multiple external stresses^34,69,70^. Plants would balance the distribution of nutrients among organs through the most efficient and preferable distribution pattern to achieve an optimal adaptive growth^71^. Studies found that trunk leaning can mitigate growth vigor and reduces leaf nitrogen content, carbohydrate output and SS content^46^. However, our study found that the contents of SS and SP were higher on the *1* side in ITT. The SS content may increase under stress because SS not only participates in osmotic regulation of cells, but also acts as a signal substance to adapt to environmental changes^71,72^, which may be related to the fact that stress affects tree growth more than photosynthesis, leading to an accumulation of SS and SP content^53,73^.

In this study, the DBH of tree trunk was larger in ITT than that in CK, showing another effect of leaning treatment, which may be the result of nutrient allocation strategy, as more resources originally will be transported to and stored in the stressed trunk. Similarly, mechanical stimulation from strong wind enables trees to acclimate the internal structure of trunk, leading to a decrement in tree height and an increment in trunk diameter^74^ .

Furthermore, this study found that the TN in both fine roots and leaves on the *0* side was larger than that on the *1* side in ITT, which was consistent with the source-sink distance of nutrient allocation strategies. When the tree trunk was used as a reference line to divide the crown, the mass of crown on the *0* side was greater than that on the *1* side, inducing more photosynthetic products on the *0* side to transport more nutrients to the roots on the same side. A similar study found that the proportion of photosynthate allocated to fine roots from the branches on the same side of *Cunninghamia lanceolata* was higher than that in the opposite side^75^.

### Adaptive strategies in architecture and allocation

Tree stability is inseparable from the architectural and functional balance of below- and above-ground. Plant morphological response to stress is related to the maintenance of plant biomechanical balance^76^, meanwhile the morphological plasticity should be determined by the biologically design with physiological monitoring mechanisms. Based on morphological and physiological traits, there should be general adaptive strategies in response to asymmetric abiotic stresses. We should comprehensively understand the relationship between biomechanical design and nutrient allocation strategies above mentioned.

On the one hand, the inverse relationship of biomass distribution of crowns to roots follows the biomechanical design strategy. According to the lever principle, the gravity load of crown on the *1* side was mainly balanced by the tension strength of the root system on the *0* side. When the tree trunk was used as a reference line to divide the crown, the mass of the crown on the *0* side was greater and can provide a lateral pulling force and resistance against gravitropism. On the other hand, the allocation of resources follows the source-sink distance of nutrient allocation strategies. The distance from leaves to the roots on the same side is generally shorter than that on the opposite side^13,41^, and the shorter pathway has lower hydraulic resistance in the xylem and lower sugar concentration gradients in the phloem^41,77,78^, which is conducive to nutrient transport. As a result, trees ultimately maintained biomechanical balance by adjusting resource allocation. In this study, due to favorable light conditions, the crown on the *0* side had a relatively higher sunlight utilization rate, and obtained more photosynthetic products which were transported to the same-sided roots. The fine roots can correspondingly obtain more resources from the crown on the same side, thus the roots on the *0* side obtained more nutrients than the opposite side, which follows the source-sink balance. Finally, the adaptive strategies become the consistent integration of biomechanical design and source-sink distance of nutrient allocation. The strategies can also be found in the asymmetric pruning test of *Cunninghamia lanceolata* that more carbon was distributed to roots from leaves on the same side than the other side, while the nitrogen content from roots showed no significant difference between the two sides of crown at 10 weeks after the root pruning treatment, and larger on the opposite side of root pruning than that on the same side at 60 weeks^13^, which may be related to that the allocation of resources initially following the source-sink distance balance, and then the biomechanical balance.

## Conclusions

The root dynamics of camphor saplings showed opposite architectural asymmetry in response to the inclined trunk treatment, following the biomechanical design strategy for tree anchorage and stability. The activities of antioxidant enzymes SOD, POD, and CAT in leaves on the *0* side were higher, while the soluble sugar and protein on the *0* side were lower than those on the *1* side. The roots on the *0* side obtained more biomass, more TN content, and greater number of fine roots to increase the tension force, while the roots on the *1* side had larger diameter, shorter length, and horizontally-oriented growth to increase compression force. The higher leaf TN on the *0* side supported more nitrogen transported to the same-sided roots, following the source-sink distance balance. This study reveals the adaptive strategies of the consistent integration of biomechanical design and source-sink distance of nutrient allocation. Based on the adaptive strategies, we can better understand the response mechanism of trees to asymmetric stress. The spatial interrelations of root-shoot architectures can be used to predict future canopy asymmetry based on the heterogeneous growth space for root system. Furthermore, the root system design can improve crown architecture related to tree anchorage and stability. In this study, however, all saplings were in the same environment, without taking into account the effects of soil and weather, as well as the magnitude and duration of dynamic forces exerted on leaves and stems. Further studies should focus on how plants unify resource allocation strategies and mechanical balance strategies which can be applied to other tree species to generalize the adaptive strategies.

## Data Availability

The data presented in this study are available on request from the corresponding author.
